# Supplementary material for: A defined clathrin-mediated trafficking pathway regulates sFLT1/VEGFR1 secretion from endothelial cells
Source: Angiogenesis. Author manuscript; Available in PMC 2024 Mar 8. (PMC10881643; doi:10.1007/s10456-023-09893-6)
Supplement: Supplemental Figures 1-6 [file NIHMS1941240-supplement-Supplemental_Figures_1-6.pdf]

SUPPLEMENTAL FIGURES AND TABLES

A defined clathrin-mediated trafficking pathway regulates sFLT1/VEGFR1 secretion from endothelial cells

Karina Kinghorn[1], Amy Gill[6], Allison Marvin[2], Renee Li[2], Kaitlyn Quigley[2], Simcha Singh[2], Michaelanthony T Gore[2], Ferdinand le Noble[5], Feilim Mac Gabhann[6], Victoria L Bautch[1,2,3,4+]

Supplemental Figures: Pages 1-7

Supplemental Movie Legends: Page 8

Supplemental Tables: Pages 9-16

SUPPLEMENTAL FIGURES

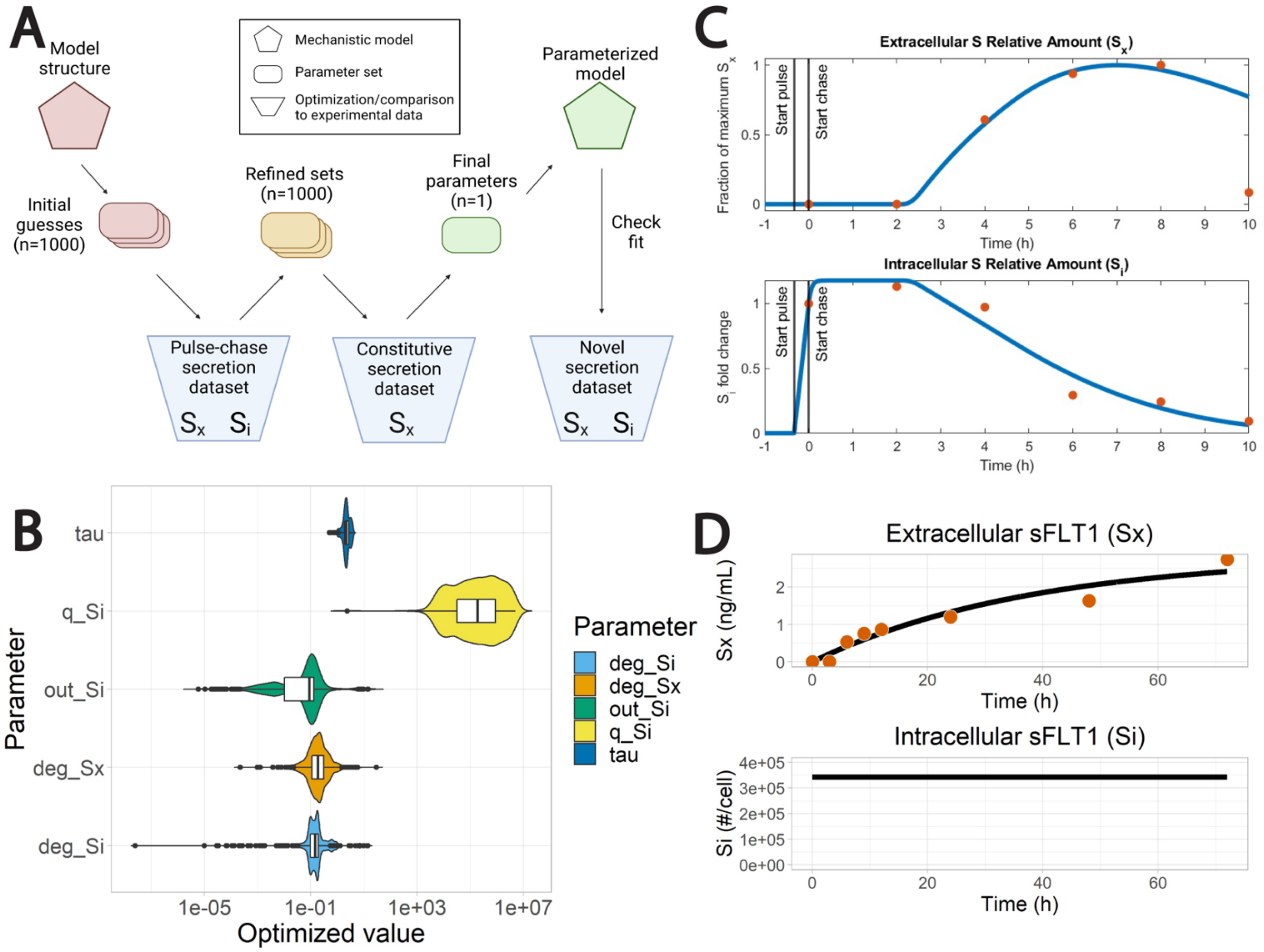

Supp. Fig. 1 Optimization of modeling parameters. (A) Optimization protocol for creating a mechanistic model for sFLT1 secretion. Model parameters were first optimized to pulse-chase secretion data [1]. The lowest cost parameter set was refined to fit absolute protein concentrations from constitutive sFLT1 secretion [2]. The final model was compared to the novel sFLT1 secretion time course from this study. Schematic created with BioRender.com. **(B)** Distribution of values for 1000 parameter sets optimized to sFLT1 pulse-chase data [1]. All parameters except q_Si are well-constrained. **(C)** Experimental data (red; [1]) and simulation with best-fit optimized parameters (blue) of intracellular and extracellular sFLT1 during pulse-chase analysis of sFLT1 secretion. **(D)** Experimental data (orange dots; [2]) and simulation with best-fit optimized parameters (black) of extracellular and intracellular sFLT1 during constitutive sFLT1 secretion.

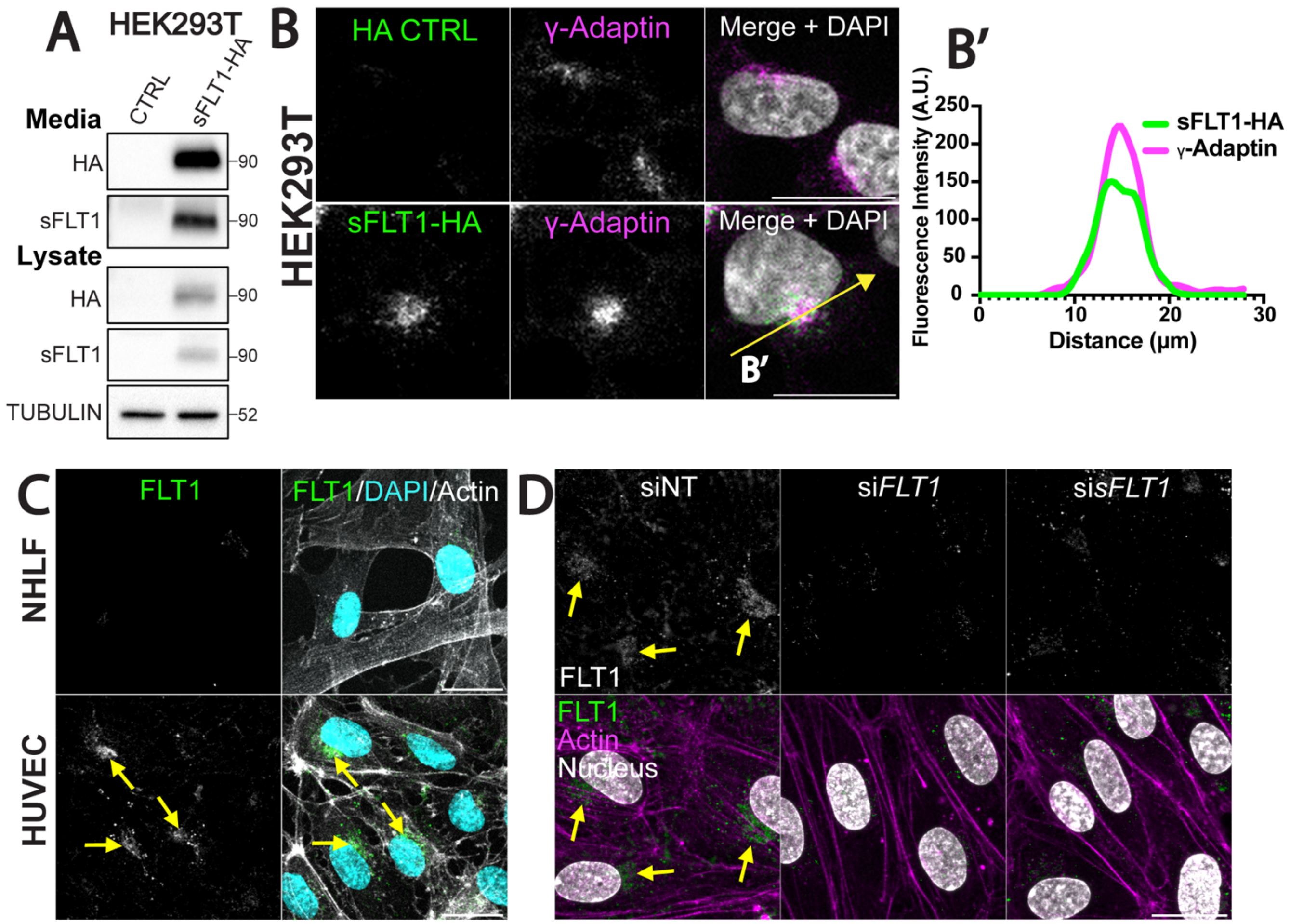

Supp. Fig. 2 sFLT1 and sFLT1-HA expression. (A) Immunoblot showing sFLT1-HA in concentrated conditioned media or cell lysates of HEK293T cells transfected with indicated DNAs and probed with indicated antibodies. Tubulin loading control. **(B)** Immunofluorescence of HEK293T cells expressing sFLT1-HA and stained for HA, γ-Adaptin (Golgi), and DAPI (nucleus). Scale bar, 20 μm. Yellow line, line scan. **(B')** Line scan of sFLT1-HA and γ-Adaptin fluorescence intensity. **(C)** Control NHLF and HUVEC immunofluorescence with indicated antibodies: FLT1, Phalloidin (actin), DAPI (nucleus). Yellow arrows: perinuclear FLT1 localization. Scale bar, 20 μm. **(D)** Immunofluorescence staining of FLT1, DAPI (nucleus), and phalloidin (actin) in HUVEC transfected with siRNAs targeting mFLT1 and sFLT1 (si*FLT1*), sFLT1 (sis*FLT1*), or non-targeting control (siNT). Yellow arrows: perinuclear FLT1 localization. Scale bar, 25 μm.

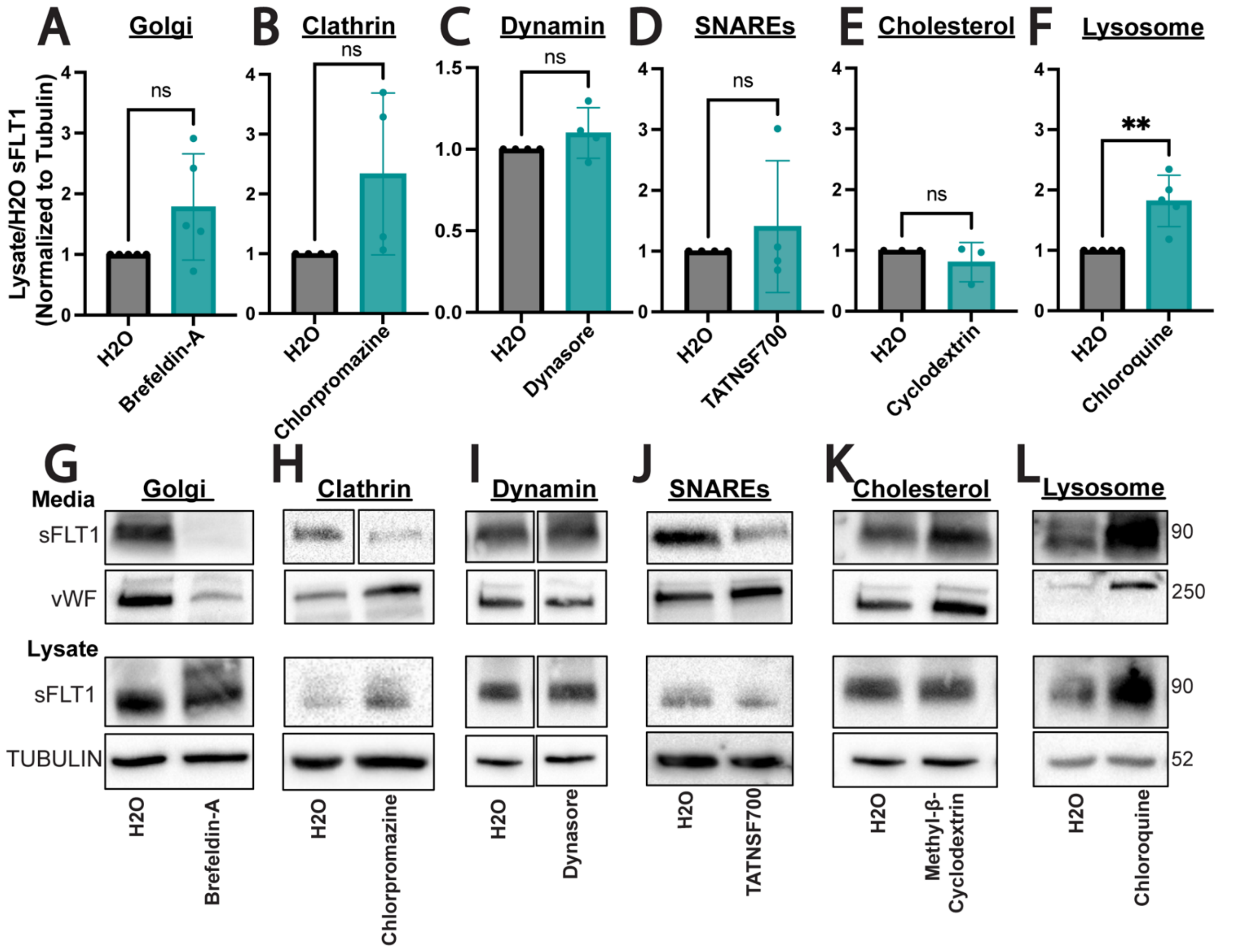

Supp. Fig. 3 Intracellular endothelial sFLT1 analysis and representative immunoblots after pharmacological inhibition. (A-F) Quantification of HUVEC lysate immunoblots treated with indicated inhibitors for 18 hr prior to collection of lysates and incubated with FLT1 antibody. Normalized to tubulin, then H_2O control. Mean +/- SD. Statistics: student's two-tailed t-test, **P<0.01, ns, not significant. **(G-L)** Representative western blots of endothelial sFLT1 and vWF in concentrated conditioned media and sFLT1 in lysates following indicated pharmacological manipulation. Tubulin loading control. Cropped bands are from the same blot.

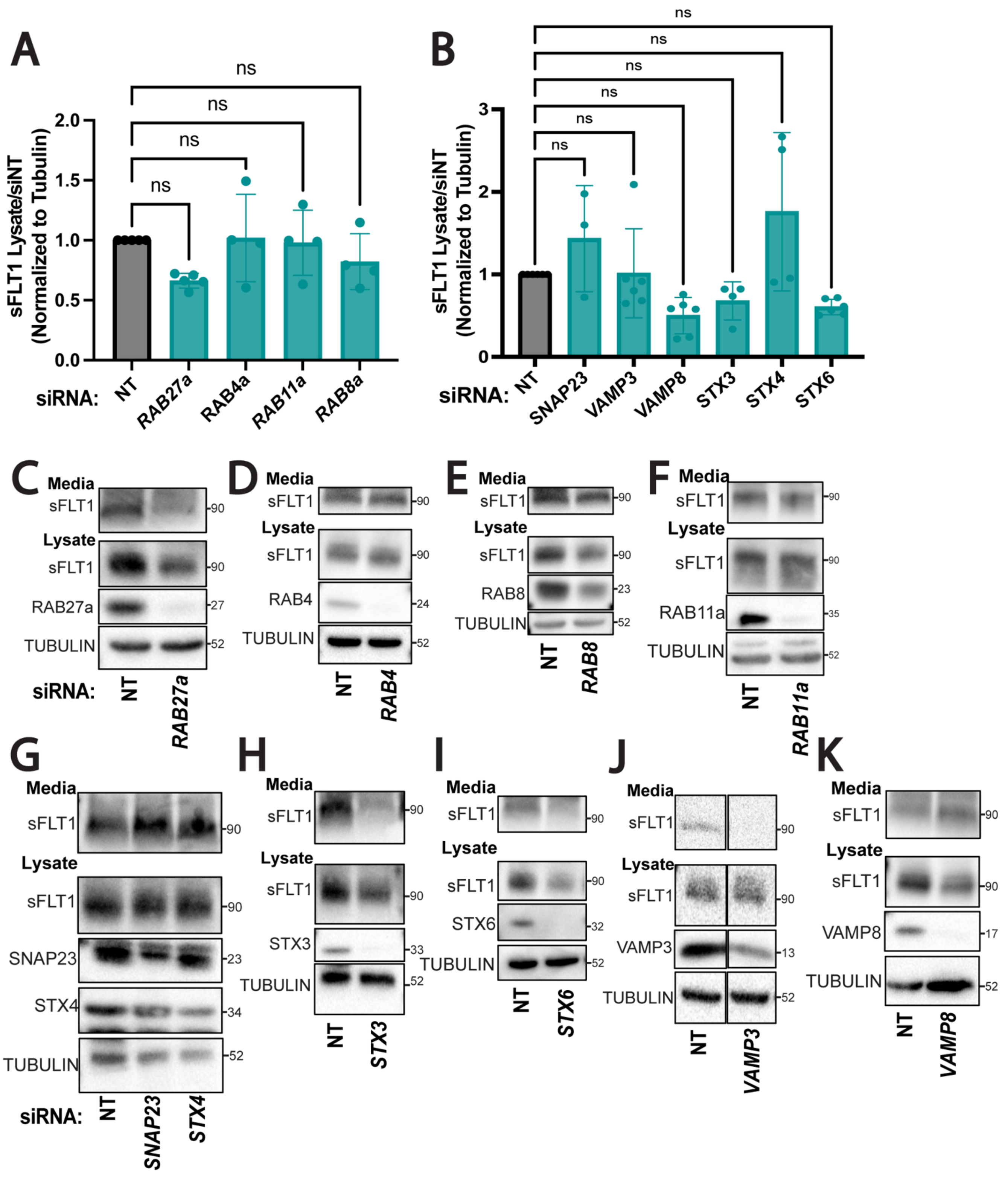

Supp. Fig. 4 Intracellular endothelial sFLT1 and representative western blots after RAB and SNARE manipulations. (A,B) Quantification HUVEC immunoblots following indicated siRNA treatments. Normalized to tubulin, then siNT. Mean +/- SD of experimental replicates shown. Statistics: student's two-tailed t-test, ns, not significant. **(C-K)** Representative western blots of endothelial sFLT1 in concentrated conditioned media and lysates following indicated siRNA treatments. Tubulin loading control. Cropped bands are from the same blot.

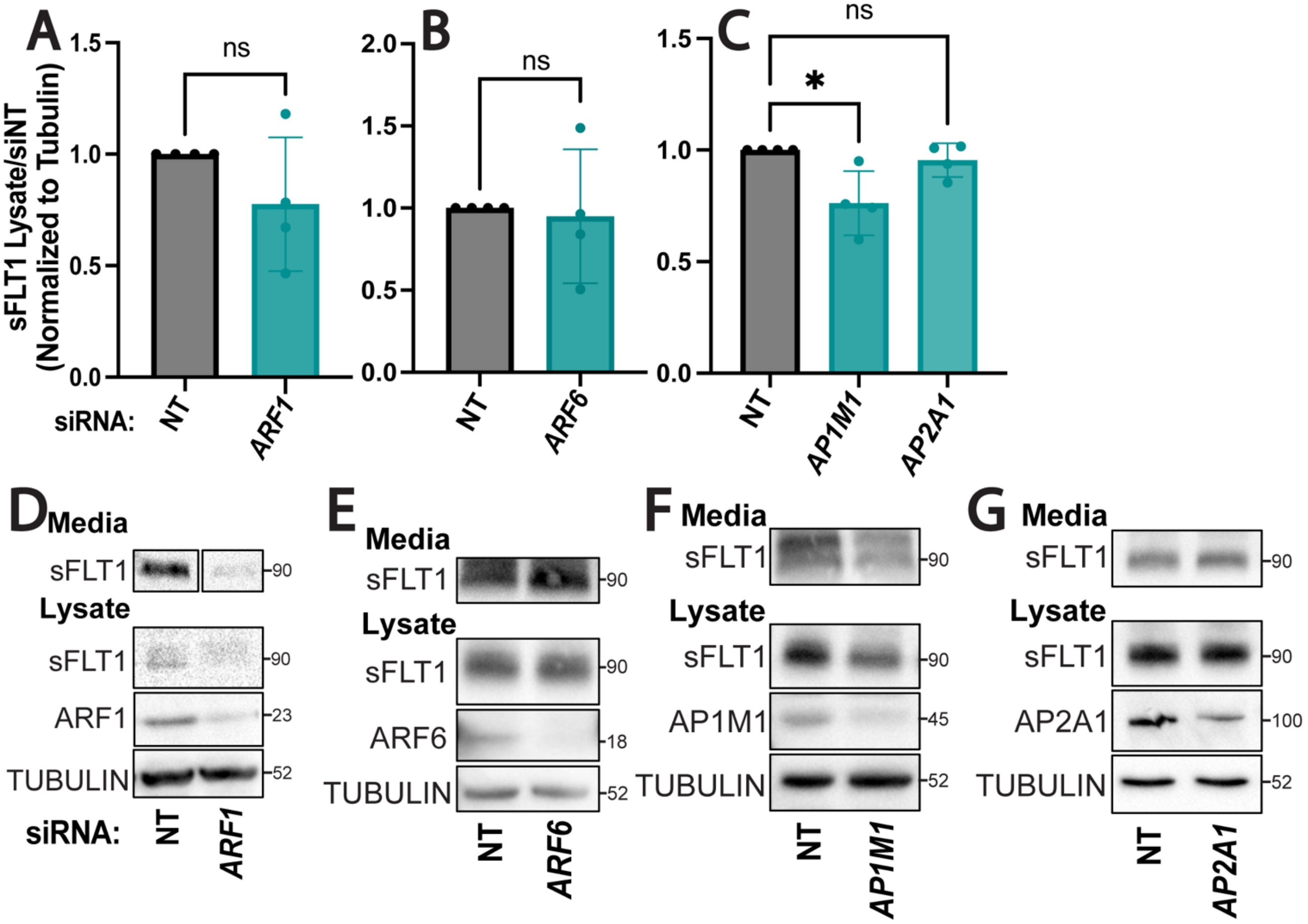

Supp. Fig. 5 Internal endothelial cell sFLT1 levels and media blots after depletion of Golgi-localized proteins. (A-C) Quantification of HUVEC immunoblots following indicated siRNA treatments. Normalized to tubulin, then siNT. Mean +/- SD of experimental replicates shown. Statistics: student's two-tailed t-test, *P<0.05, ns, not significant. **(D-G)** Representative western blots of endothelial sFLT1 in concentrated conditioned media and lysates following indicated siRNA treatments. Tubulin loading control. Cropped bands are from the same blot.

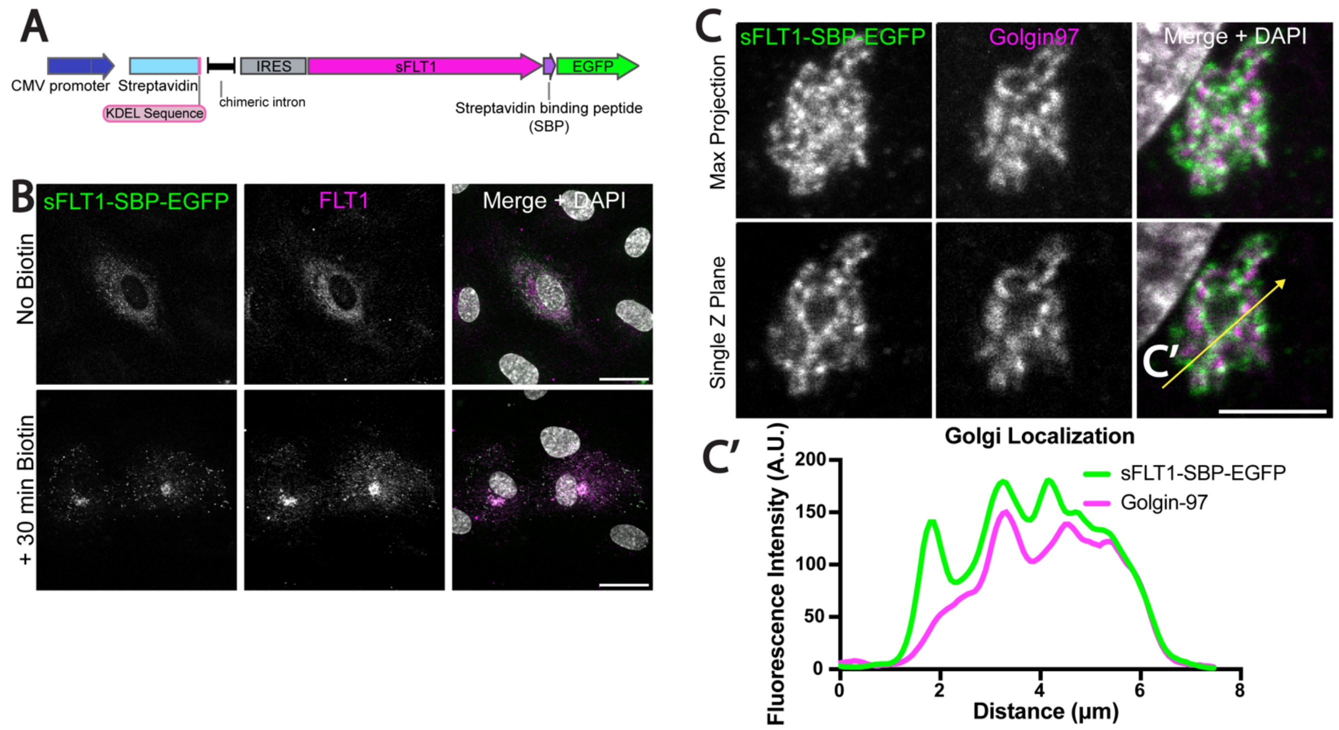

Supp. Fig. 6 Tagged sFLT1 trafficking in HUVEC. (A) Map of tagged sFLT1 (sFLT1-SBP-EGFP) construct. **(B)** Immunofluorescence staining of tagged sFLT1 in HUVEC with FLT1 antibody and DAPI +/- 30 min biotin incubation. Scale bar, 25 μm. **(C)** Immunofluorescence staining of tagged sFLT1 + HUVEC with Golgin-97 (trans-Golgi) and DAPI 30 min post-biotin addition. Scale bar, 10 μm. Yellow line, line scan. **(C')** Line scan of tagged sFLT1 and Golgin-97 intensity in single Z plane.

SUPPLEMENTAL MOVIES

Movie 1. Live-cell trafficking of tagged sFLT1 in HUVEC. Time-lapse imaging of HUVEC transiently expressing tagged sFLT1 (sFLT1-SBP-EGFP). Max Z projection is shown. Images acquired every 6.58 seconds from 10-45 min after biotin addition. Scale bar, 10 μm. Corresponds to Figure 9 B-C.

Movie 2. Tagged sFLT1 vesicle trafficking in vehicle control. Time-lapse imaging of HUVEC transiently expressing tagged sFLT1 (sFLT1-SBP-EGFP) and treated with H2O control. Max Z projection is shown. Images acquired every 6.58 seconds from 30-50 min after biotin addition; vesicles marked at 33.33 min time stamp. Scale bar, 10 μm.

Movie 3. Tagged sFLT1 vesicle trafficking after chlorpromazine treatment. Time-lapse imaging of HUVEC transiently expressing tagged sFLT1 (sFLT1-SBP-EGFP) and treated with chlorpromazine for 4 hr. Max Z projection is shown. Images acquired every 6.58 seconds from 30-50 min after biotin addition; vesicles marked at 44.45 min time stamp. Scale bar, 10 μm.

SUPPLEMENTAL TABLES

Supplementary Table S1. Delay differential equations describing intracellular and extracellular sFLT1 amount over time in the mechanistic model. Parameter descriptions and optimal values found in Supplementary Table S4. In the S_x equation, a conversion factor is applied to the S_i term to obtain consistent units.

Species	Description	Units	Rate of change equation
S_i	Intracellular sFLT1	#/cell	$\frac{dS_i(t)}{dt} = k_{q\_Si} - k_{out\_Si}S_i(t-\tau) - k_{deg\_Si}S_i(t)$
S_x	Extracellular sFLT1	ng/mL	$\frac{dS_x(t)}{dt} = k_{out\_Si}S_i(t-\tau) - k_{deg\_Sx}S_x(t)$

Supplementary Table S2. Characteristics of sFLT1 datasets used to build the mechanistic model.

Sx, extracellular sFLT1; Si, intracellular sFLT1; +, experimentally measured; -, not measured.

* Sx converted to fraction of maximum amount for optimization.

† Identification of a system of equations and estimation of secretion parameters.

‡ Estimation of biologically relevant production parameter values.

Reference	Experiment	S_x	S_i	Measurement	Units	Time points (h)	Rationale
Jung et al., 2012 [1]	Pulse-chase	+	+	autoradiography	Fold change*	0, 2, 4, 6, 8, 10	†
Hornig et al., 2000 [2]	Constitutive secretion	+	-	ELISA	ng/mL	0, 3, 6, 9, 12, 24, 48, 72	‡

Supplementary Table S3. Input parameters for mechanistic computational model.

Variable	Units	Value	Reference
Number of cells	cells	5000	Hornig et al., 2000 [2]
Media volume	mL	0.3	Hornig et al., 2000 [2]; standard media volume for 48-well plate

Supplementary Table S4. Optimized parameters for mechanistic computational model.

Rate constant	Process	Units	Value
k_{q_Si}	sFLT1 synthesis	(#/cell)/h	6.01×10^4
k_{out_Si}	sFLT1 secretion	1/h	$.0878$
k_{deg_Si}	Intracellular sFLT1 degradation	1/h	$.0873$
k_{deg_Sx}	Extracellular sFLT1 degradation	1/h	$.0264$
τ	sFLT1 secretion delay	h	2.47

Supplementary Table S5. Reported secretion rates of various proteins. † Using the following assumptions: (a) secretion rates were assumed constant when reported over a longer time span (e.g. #/cell/day) and (b) cell count was assumed constant when proliferation was not incorporated in the original rate estimate. ‡ Calculated using molecular weight and Avogadro's number. Abbreviations: ASC = adipocyte stromal cell, HUVEC = human umbilical vein endothelial cell, PBMC = peripheral blood mononuclear cell.

Protein	Cell type	Species	Molecular weight (kDa)	Secretion rate (g/cell/h) †	Secretion rate (#/cell/h) ‡	References
IgM	plasma cell	trout	800	1.39×10^{-10}	1.05×10^{8}	Bromage et al., 2009 [3]; Bromage et al., 2004 [4]
IgE	plasma cell	human	150	6.25×10^{-12}	2.51×10^{7}	Werner-Favre et al., 1993 [5]
IgA	plasma cell	human	150	5.67×10^{-12}	2.27×10^{7}	Werner-Favre et al., 1993 [5]
IgG	plasma cell	human	150	4.67×10^{-12}	1.87×10^{7}	Werner-Favre et al., 1993 [5]
IgG	plasma cell	human	150	$2 - 29 \times 10^{-13}$	$0.8 - 1.2 \times 10^{7}$	Janossy et al., 1977 [6]
IgM	plasma cell	human	950	1.14×10^{-11}	7.2×10^{6}	Werner-Favre et al., 1993 [5]
IgG	plasma cell	human	150	$2 - 14 \times 10^{-13}$	$0.8 - 5.7 \times 10^{6}$	Salmon and Smith, 1970 [7]
albumin	liver	rat	65	1.56×10^{-13}	1.44×10^{6}	Chen and Redman, 1977 [8]
IgM	plasma cell	human	950	$2 - 17 \times 10^{-13}$	$0.13 - 1.0 \times 10^{6}$	Janossy et al., 1977 [6]
vWF	HUVEC	human	250	2.92×10^{-14}	7.03×10^{4}	Hakkert et al., 1992 [9]
IFNγ	Spleen	mouse	20	7.98×10^{-16}	4.80×10^{4}	Favre et al., 1997 [10]
TGF- β	ASC	human	25	1.73×10^{-17}	4.17×10^{3}	Rehman et al., 2004 [11]
IL-6	PBMC	human	21	$7.8 - 10 \times 10^{-17}$	$2.2 - 2.9 \times 10^{3}$	O'Mahony et al., 1998 [12]
TNF-α	PBMC	human	17	$3.5 - 5.4 \times 10^{-17}$	$1.2 - 1.9 \times 10^{3}$	O'Mahony et al., 1998 [12]
HGF	ASC	human	80	1.71×10^{-16}	1.28×10^{3}	Rehman et al., 2004 [11]
VEGF (hypoxia)	ASC	human	40	8.31×10^{-17}	1.25×10^{3}	Rehman et al., 2004 [11]
IL-1β	PBMC	human	17	$2.1 - 2.2 \times 10^{-17}$	$7.4 - 7.9 \times 10^{2}$	O'Mahony et al., 1998 [12]
VEGF (normoxia)	ASC	human	40	1.67×10^{-17}	2.51×10^{2}	Rehman et al., 2004 [11]
bFGF	ASC	human	22	1.72×10^{-17}	4.71×10^{1}	Rehman et al., 2004 [11]
GM-CSF	ASC	human	15	1.17×10^{-17}	4.68×10^{1}	Rehman et al., 2004 [11]

Key Resources

Supplemental Table S6. Pharmacological Inhibitors.

Inhibitor	Final Concentration	Stock Solution	Company	Target
Brefeldin-A	1 mg/mL	5 mg/mL in DMSO	Biolegend (420601)	Blocks the GEF of ARF1 and leads to Golgi collapse
Chlorpromazine	10 μM (HUVEC), 100 μM (fish)	10 mM in H2O	Sigma (C8138)	Prevents assembly and disassembly of clathrin lattices
Dynasore	5 μM	10 mM in DMSO	Santa Cruz (sc-202592)	Inhibits the GTPase of dynamin
TATNSF-700	1 μM	1 mM in H2O	AnaSpec (AS-62238)	Competes for the NSF binding and blocks SNARE disassembly
Methyl-B-Cyclodextrin	1mM	10 mM in H2O	Sigma (C4555)	Depletes membranes of cholesterol
Chloroquine	10 μg/mL	10 mg/mL in H2O	Sigma (C6628)	Elevates lysosomal pH to block degradation

Supplemental Table S7. siRNAs.

siRNAs	Catalog #	Company	pmol/10^{cm2}	Target Sequence (5'--> 3')
FLT1-1	4392420 (s5287)	ThermoFisher	200 pmol	GGUGAGUAAGGAAAGCGAAtt
FLT1-2	sc-29319	Santa Cruz	200 pmol	N/A
sFLT1	4390827 (custom)	ThermoFisher	200 pmol	AAGGCUGUUUUCUCUCGGAUU
ARF1-1	4390824 (s1551)	ThermoFisher	200 pmol	CCAUAGGCUUCAACGUGGAtt
ARF1-2	L-011580-00-0005	Dharmacon	200 pmol	UGACAGAGAGCGUGUGAAC, CGGCCGAGAUCACAGACAA, ACGAUCCUCUACAAGCUUA, GAACCAGAAGUGAACGCGA
STX6-1	4392420 (s19959)	ThermoFisher	200 pmol	GCAACUGAAUUGAGUAUAAtt
STX6-2	4392420 (s19958)	ThermoFisher	200 pmol	CCAACGAGCUGAGAAAUAAtt
AP1M1	4392420 (s17032)	ThermoFisher	200 pmol	ACAACUUUGUUAUCAUCUAtt
AP2A1	4390824 (s184)	ThermoFisher	200 pmol	GCCGAUGAGUUGCUGAAUAtt
RAB27a-1	4390824 (s11695)	ThermoFisher	200 pmol	GGAAGACCAGUGUACUUUAtt
RAB27a-2	4390824 (s11693)	ThermoFisher	200 pmol	GCCUCUACGGAUCAGUUAAtt
RAB4	439084 (s11675)	ThermoFisher	200 pmol	GGUCCGUGACGAGAAGUUAtt
RAB11a-1	sc-36340	Santa Cruz	200 pmol	N/A
RAB11a-2	4390824 (s16702)	ThermoFisher	200 pmol	CAACAAUGUGGUUCCUAUUtt
RAB8a	4390824 (s8681)	ThermoFisher	200 pmol	CUUUAAAAUUAGGACCAUAtt
ARF6-1	4390824 (s1567)	ThermoFisher	200 pmol	CCAAGGUCUCAUCUUCGUAtt
ARF6-2	4390824 (s1565)	ThermoFisher	200 pmol	CUCUCAUCUUCGUAGUGGAtt
SNAP23-1	sc-41308	Santa Cruz	200 pmol	N/A
SNAP23-2	4392420 (s16709)	ThermoFisher	200 pmol	GGAACAACUAAACCGCAUAtt
STX4-1	sc-36590	Santa Cruz	200 pmol	N/A
STX4-2	4392420 (s13597)	ThermoFisher	200 pmol	UGAUCAAUCGGAUUGAGAAtt
STX3-1	sc-616132	Santa Cruz	200 pmol	N/A
STX3-2	4392420 (s13592)	ThermoFisher	200 pmol	GGCACGAGAUGAAACGAAAtt
VAMP3-1	sc-41338	Santa Cruz	200 pmol	N/A
VAMP3-2	4392420 (s17856)	ThermoFisher	200 pmol	CGGGAUUACUGUUCUGGUUtt

VAMP8-1	sc-41300	Santa Cruz	200 pmol	N/A
VAMP8-2	4392420 (s16522)	ThermoFisher	200 pmol	GGAGUUAAGAAUAUUAUGAtt
Non-targeting-1	4390844	ThermoFisher	200 pmol	N/A
Non-targeting-2	4390847	ThermoFisher	200 pmol	N/A
Non-targeting-3	sc-37007	Santa Cruz	200 pmol	N/A

Supplemental Table S8. Primary Antibodies.

Target	Host Species	Company	Catalog Number	Western Dilution	IF Dilution
FLT1	goat	RnD	AF321	1:10,000	1:500
VWF	mouse	ThermoFisher	MA5-14029	1:500	1:1000
HA	rabbit	Cell Signaling	3724S	1:1,000	1:500
HA	mouse	Biolegend	901502	1:500	1:500
GM130 (Cis-Golgi)	rabbit	abcam	ab52649	1:1,2000	1:500
Golgin-97 (Trans-Golgi)	mouse	ThermoFisher	14-9767-80	N/A	1:500
STX6 (Syntaxin 6, Trans-Golgi)	rabbit	Cell Signlaing	2869s	1:500	1:250
γ-Adaptin (adaptor protein 1 γ subunit)	rabbit	abcam	ab220251	N/A	1:500
AP1M1 (adaptor protein 1, μ1 subunit)	rabbit	Proteintech	12112-1-AP	1:1000	N/A
AP2A1 (adaptor protein 2, α1 subunit)	mouse	ThermoFisher	MA3-061	1:2000	N/A
Clathrin Heavy Chain	mouse	ThermoFisher	MA1-065	N/A	1:500
Tubulin	rabbit	Cell Signaling	2144S	1:50,000	N/A
Tubulin	mouse	Cell Signaling	3873S	1:100,000	N/A
GAPDH	mouse	Cell Signaling	97166s	1:50,000	N/A
ZO1	mouse	ThermoFisher	33-9100	1:1000	N/A
EEA1	mouse	BD Biosciences	610456	1:2000	N/A
Calnexin	mouse	ThermoFisher	MA3-027	1:1000	N/A
RAB27a	rabbit	Cell Signaling	95394S	1:2000	N/A
ARF1	rabbit	abcam	ab76082	1:1000	N/A
ARF6	rabbit	abcam	ab77581	1:1000	N/A
SNAP23	rabbit	abcam	ab3340	1:2000	N/A
STX3	rabbit	abcam	ab133750	1:2000	N/A
STX4	rabbit	abcam	ab184545	1:1000	N/A
VAMP3 (Cellubrevin)	rabbit	abcam	ab43080	1:5000	N/A
VAMP8	rabbit	abcam	ab76021	1:10,000	N/A
RAB4	mouse	ThermoFisher	MA5-17161	1:1000	N/A
RAB11	rabbit	abcam	ab65200	1:1000	N/A
VE-Cadherin	rabbit	Cell Signaling	2500s	N/A	1:500

Supplemental Table S9. Secondary Antibodies.

Antibody	Catalog Number	Company	Western Dilution	IF Dilution
Donkey anti-goat IgG HRP	PA1-28664	ThermoFisher	1:20,000	N/A
Donkey anti-Rabbit IgG (H+L) Highly Cross-Adsorbed Secondary Antibody, HRP	A16035	ThermoFisher	1:10,000	N/A
Donkey anti-Mouse IgG (H+L) Highly Cross-Adsorbed Secondary Antibody, HRP	A16011	ThermoFisher	1:10,000	N/A
Goat anti-Rabbit HRP	31460	ThermoFisher	1:10,000	N/A
Goat anti-Mouse HRP	31430	ThermoFisher	1:10,000	N/A
Donkey anti Goat IgG (H+L) Highly Cross Absorbed Secondary Antibody, Alexa Fluor 488	A-11055	ThermoFisher	N/A	1:1,000
Donkey anti Rabbit IgG (H+L) Highly Cross Absorbed Secondary Antibody, Alexa Fluor 488	A-21206	ThermoFisher	N/A	1:1,000
Donkey anti Mouse IgG (H+L) Highly Cross Absorbed Secondary Antibody, Alexa Fluor 488	A-21202	ThermoFisher	N/A	1:1,000
Donkey anti Rabbit IgG (H+L) Highly Cross Absorbed Secondary Antibody, Alexa Fluor 594	A-21207	ThermoFisher	N/A	1:1,000
Donkey anti Mouse IgG (H+L) Highly Cross Absorbed Secondary Antibody, Alexa Fluor 594	A-21203	ThermoFisher	N/A	1:1,000
Donkey anti Goat IgG (H+L) Highly Cross Absorbed Secondary Antibody, Alexa Fluor 647	A32849	ThermoFisher	N/A	1:1,000
Donkey anti Rabbit IgG (H+L) Highly Cross Absorbed Secondary Antibody, Alexa Fluor 647	A-31573	ThermoFisher	N/A	1:1,000
Donkey anti Mouse IgG (H+L) Highly Cross Absorbed Secondary Antibody, Alexa Fluor 647	A-31571	ThermoFisher	N/A	1:1,000
Alexa Fluor 594 Phalloidin	A12381	ThermoFisher	N/A	1:500
Alexa Fluor 647 Phalloidin	A22287	ThermoFisher	N/A	1:500
DAPI	10236276001	Sigma	N/A	1:1,000

REFERENCES

1. Jung J-J, Tiwari A, Inamdar SM, Thomas CP, Goel A, Choudhury A (2012) Secretion of Soluble Vascular Endothelial Growth Factor Receptor 1 (sVEGFR1/sFlt1) Requires Arf1, Arf6, and Rab11 GTPases. PLoS ONE 7 (9):e44572. doi:10.1371/journal.pone.0044572
2. Hornig C, Barleon B, Ahmad S, Vuorela P, Ahmed A, Weich HA (2000) Release and Complex Formation of Soluble VEGFR-1 from Endothelial Cells and Biological Fluids. Lab Invest 80 (4):443-454. doi:10.1038/labinvest.3780050
3. Bromage E, Stephens R, Hassoun L (2009) The third dimension of ELISPOTs: quantifying antibody secretion from individual plasma cells. J Immunol Methods 346 (1-2):75-79. doi:10.1016/j.jim.2009.05.005
4. Bromage ES, Ye J, Owens L, Kaattari IM, Kaattari SL (2004) Use of staphylococcal protein A in the analysis of teleost immunoglobulin structural diversity. Dev Comp Immunol 28 (7-8):803-814. doi:10.1016/j.dci.2003.12.001
5. Werner-Favre C, Matthes T, Barnet M, Zubler RH (1993) High IgE secretion capacity of human plasma cells. Eur J Immunol 23 (8):2038-2040. doi:10.1002/eji.1830230849
6. Janossy G, Gomez de la Concha E, Luquetti A, Snajdr MJ, Waxdal MJ, Platts-Mills TA (1977) T-cell regulation of immunoglobulin synthesis and proliferation in pokeweed (Pa-1)-stimulated human lymphocyte cultures. Scand J Immunol 6 (1-2):109-123. doi:10.1111/j.1365-3083.1977.tb00326.x
7. Salmon SE, Smith BA (1970) Immunoglobulin synthesis and total body tumor cell number in IgG multiple myeloma. J Clin Invest 49 (6):1114-1121. doi:10.1172/jci106327
8. Chen LL, Redman CM (1977) Synthesis and secretion of albumin by a synchronized rat hepatoma cell line. Biochim Biophys Acta 479 (1):53-68. doi:10.1016/0005-2787(77)90125-3

9. Hakkert BC, Rentenaar JM, van Mourik JA (1992) Monocytes enhance endothelial von Willebrand factor release and prostacyclin production with different kinetics and dependency on intercellular contact between these two cell types. Br J Haematol 80 (4):495-503. doi:10.1111/j.1365-2141.1992.tb04563.x

10. Favre N, Bordmann G, Rudin W (1997) Comparison of cytokine measurements using ELISA, ELISPOT and semi-quantitative RT-PCR. J Immunol Methods 204 (1):57-66. doi:10.1016/s0022-1759(97)00033-1

11. Rehman J, Traktuev D, Li J, Merfeld-Clauss S, Temm-Grove CJ, Bovenkerk JE, Pell CL, Johnstone BH, Considine RV, March KL (2004) Secretion of angiogenic and antiapoptotic factors by human adipose stromal cells. Circulation 109 (10):1292-1298. doi:10.1161/01.Cir.0000121425.42966.F1

12. O'Mahony L, Holland J, Jackson J, Feighery C, Hennessy TP, Mealy K (1998) Quantitative intracellular cytokine measurement: age-related changes in proinflammatory cytokine production. Clin Exp Immunol 113 (2):213-219. doi:10.1046/j.1365-2249.1998.00641.x
